# Electrodeposited Cu/MWCNT composite-film: a potential current collector of silicon-based negative-electrodes for Li-Ion batteries†

**DOI:** 10.1039/c9ra03000j

**Published:** 2019-07-15

**Authors:** Masahiro Shimizu, Tomonari Ohnuki, Takayuki Ogasawara, Taketoshi Banno, Susumu Arai

**Affiliations:** Department of Materials Chemistry, Faculty of Engineering, Shinshu University 4-17-1 Wakasato Nagano 380-8553 Japan shimizu@shinshu-u.ac.jp araisun@shinshu-u.ac.jp +81-26-269-5627 +81-26-269-5432 +81-26-269-5627 +81-26-269-5413; Institute of Carbon Science and Technology, Faculty of Engineering, Shinshu University 4-17-1 Wakasato Nagano 380-8553 Japan

## Abstract

With the aim of developing the potential high theoretical capacity of Si as a negative electrode material for Li-ion batteries, a new type of composite current collector in which multi-walled carbon nanotubes (MWCNTs) are immobilized on a Cu surface was developed using an electroplating technique. For the Si electrode with a flat-Cu substrate, voltage plateaus related to the stepwise electrochemical lithiation were observed below 0.27 V (*vs.* Li/Li^+^), whereas the Cu/MWCNT substrate distinctly decreased the overvoltage to enhance charge/discharge capacities to approximately 1.6 times that obtained in the flat-Cu system. Field-emission scanning microscopy revealed that MWCNTs immobilized on the Cu surface extended inside the active material layer. Adhesion strength between the substrate and electrode mixture layer was reinforced by MWCNTs to increase the reversibility of change in electrode thickness before and after cycling: the expansion ratio was 200% and 134% for flat-Cu and Cu/MWCNT systems, respectively. Electrochemical impedance analysis demonstrated that MWCNTs served as an electron conduction pathway inside the electrode. By controlling the upper cutoff voltage from 2.0 V to 0.5 V, synergetic effects including improved adhesion strength and a more developed conduction pathway became noticeable: a reversible capacity of 1100 mA h g^−1^ with 64% capacity retention was achieved even after the 100th cycle. The results indicate that the Cu/MWCNT is a promising current collector for expansion/contraction-type active materials for rechargeable batteries.

## Abstract 2

To develop the potential high theoretical capacity of Si as a negative electrode material for Li-ion batteries, a new type of composite current collector in which carbon nanotubes (CNTs) are immobilized on a Cu surface was developed using an electroplating technique.

## Graphical abstract

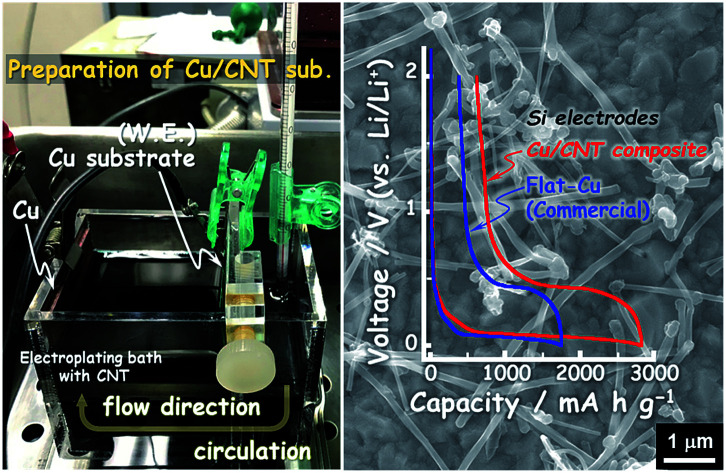

## Introduction

**Fig. 1:**
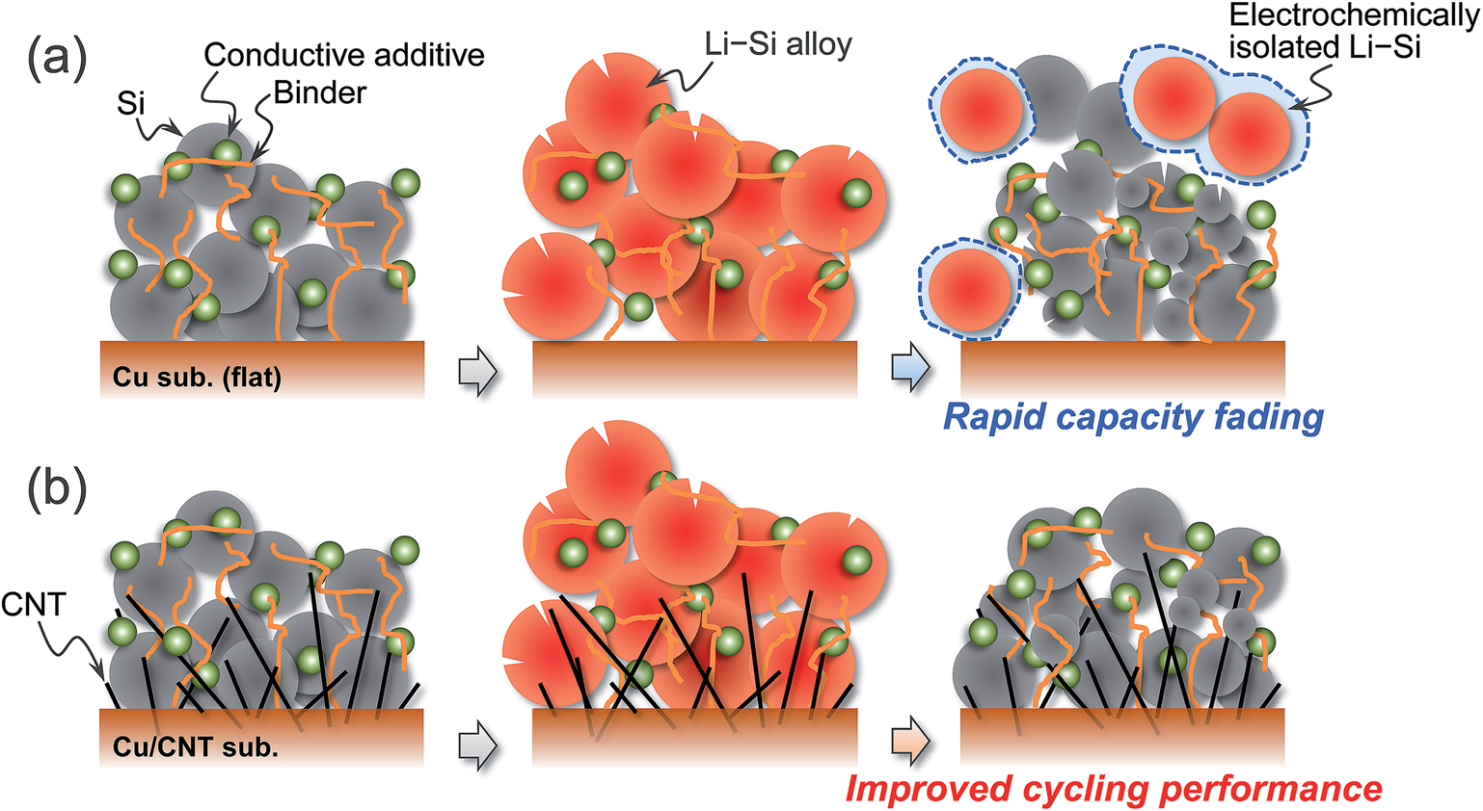
(a) Schematic illustration of deterioration mechanism of a Si electrode using typical current collector during charge/discharge cycling. (b) Proposed mechanism of improved performance of a Si electrode with Cu/CNT composite substrate. It is expected that CNTs play a key role in keeping an active material inside an electrode during significant expansion and contraction of Si (Li–Si alloying/dealloying reactions).

Lithium ion batteries are becoming indispensable for human beings to live comfortably and have been expanded to large scale applications such as electric vehicles and power storage units, both vital in efforts to achieve a low carbon society in recent years.^1^ Nevertheless, demand for the enhancement of their energy densities has been greatly increasing: especially for electric vehicles, it is required to achieve a target value of 500 W h kg^−1^ or more for extending driving distance.^2,3^ In order to satisfy these requirements, we get to face the challenging problem of improving the capacity of positive and negative electrode materials and establishing high voltage operation.^4–7^ Among negative electrode materials on group 14 elements attracting much attention,^2,8^ Si is expected to be a promising active material due to its high theoretical capacity of 3580 mA h g^−1^ (Li_15_Si_4_)^9–12^ originating from its electrochemical lithiation and delithiation. However, the capacity is inevitably accompanied by an extremely large volume change (Δ280%), causing pulverization followed by electrical isolation. From a macroscopic point of view, the detachment of the active material layer and crack generation are induced by the above unfavorable phenomena and thereby result in the mechanical disintegration of the electrode leading to capacity fading.^13^ Furthermore, the volume change breaks surface layers on Si particles induced by decomposition of the electrolyte and the reconstructed layers become thicker. The resulting surface layers reduce electronic contact between Si particles and thereby make it difficult to retain a high capacity for long cycles.^14,15^ Excluding the modification of the active material itself such as introducing space accommodating volume expansion,^16,17^ coating a carbon matrix,^18,19^ and microparticulation,^20,21^ the development of a binder and a roughened current collector improving adhesion between Si particles and between the substrate and active material layer is an effective approach. As for studies on current collectors, a roughening surface is usually used, and most of them are for film electrodes prepared by sputtering or electrodeposition.^22–25^ Osaka and Momma *et al.* modified a Cu substrate surface with a carbon nanotube (CNT) using electrophoretic deposition and applied it to electrodeposited Si–O–C composites. The CNTs anchor layer increased adhesion strength between the composite and substrate and resulted in improved cycling performance.^26^ With regards to the slurry-type electrode, as a top-down approach, a tensile-strength clad (Ni/Cu/Ni) foil with a roughening surface etched with an acid solution^27^ and a roughened substrate with bundles of metallic Cu formed with electrochemically etching followed by heat treatment^28^ were reported by Kataoka *et al.* and Roué *et al.*, respectively. Lee *et al.* applied rough Cu current collectors to Si electrodes in flexible batteries.^29^ In another study, they improved adhesion strength between the Si composite electrode and Cu current collector by coating polydopamine onto the Cu surface and this resulted in mitigated capacity decline.^30^ Considering manufacturing processes and their complications, roughening is preferable as a one-step and a bottom-up approach. Recently, we succeeded in embedding CNT on a Cu-film surface using an electroplating technique.^31^ Because the CNTs are immobilized in the film, we conceived the strategy of applying the Cu/CNT composite substrate to a current collector for a Si electrode undergoing significant expansion/contraction during the charge/discharge process. It is expected that CNTs extending inside an electrode mixture has not only an anchor effect but also an electron conduction pathway to improve cycle stability (Fig. 1). In the present study, we attempted to develop and control the roughened surface morphology of Cu/CNT composite substrates and to address disadvantages of Si electrodes. Herein, we report a new type of roughening method and the applicability of a Cu/CNT substrate as a current collector.

## Experimental

MWCNT (5 g L^−1^, VGCF; SHOWA DENKO K.K.) was added to an acid-based electroplating bath consisting of 0.85 mol dm^−3^ (M) CuSO_4_ + 0.55 M H_2_SO_4_ with 0.02 mM poly(acrylic acid) (PAA; average molecular weight: 5000) as the dispersant for MWCNT. After stirring and ultrasonic homogenizer treatment, the bath was cooled to 15 °C and kept at that temperature. Immobilization of MWCNT on a Cu substrate (7 × 6.7 cm^2^) was carried out with an electroplating method for 1 μm thickness (2.7 C cm^−2^) under a constant current density of 5 mA cm^−2^ at 15 °C. For promoting co-deposition of MWCNTs, the electroplating bath was put through an electrolyzer-using circulating system. The crystal structure and surface/cross-sectional morphologies of the obtained Cu/MWCNT composite substrate were characterized by X-ray diffraction (XRD; SmartLab, Rigaku), Raman spectroscopy (T64000 Advanced Research Raman System, HORIBA Ltd.) using the 532 nm line of a Nd:YAG laser, confocal laser scanning microscopy (CLSM, VK-8510; Keyence), and field-emission scanning electron microscopy (FE-SEM, JSM-7000F; JEOL Co., Ltd.). Si powder (diameter: 0.5–1 μm), Ketjen black (KB), and sodium carboxymethylcellulose (CMC) with a weight ratio of 70/20/10 wt% were mixed with an acid aqueous solution (pH 3) adjusted with potassium hydroxide and citric acid.^32^ The working electrode was prepared by casting the slurry on the composite substrate followed by drying in a vacuum at 80 °C for 30 min. The loading mass of the active material is approximately 0.8 mg cm^−2^. The weight of the active material on the substrate was measured to an accuracy of 1 μg with an ultramicrobalance (XP6V; METTLER TOLEDO). Then, the electrode was incorporated into a 2032-type coin cell which includes an Li metal foil (99.90%; Rare Metallic) as the counter electrode, a glass fiber separator, and an electrolyte of 1.0 M lithium hexafluorophosphate (LiPF_6_) dissolved in a mixture of solvents (50/50 vol%) of ethylene carbonate (EC) and diethyl carbonate (DEC) with 5 vol% fluoroethylene carbonate (FEC). Galvanostatic charge–discharge tests were conducted using an electrochemical measurement system (HJ-1001 SM8A; Hokuto Denko Co., Ltd.) in the voltage range between 0.005 and 2.000 V (*vs.* Li/Li^+^) at room temperature.

## Results and discussion

**Fig. 2:**
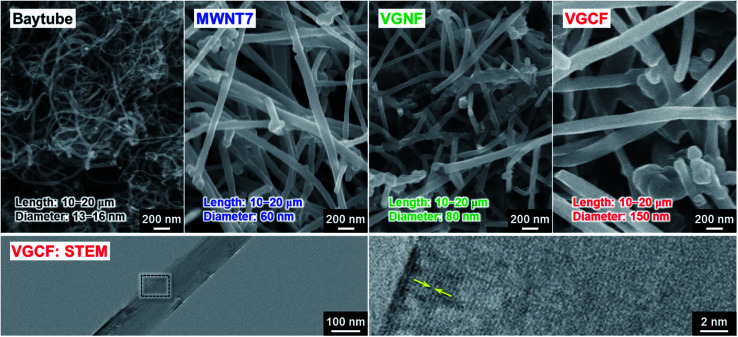
FE-SEM and STEM images of several multi-walled carbon nanotubes (MWCNTs). Diameters of Baytube (Bayer MaterialScience), MWNT7 (Hodogaya Chemical Co., Ltd.), VGNF, and VGCF (SHOWA DENKO K.K.) are 15 nm, 60 nm, 80 nm, and 150 nm, respectively.

**Fig. 3:**
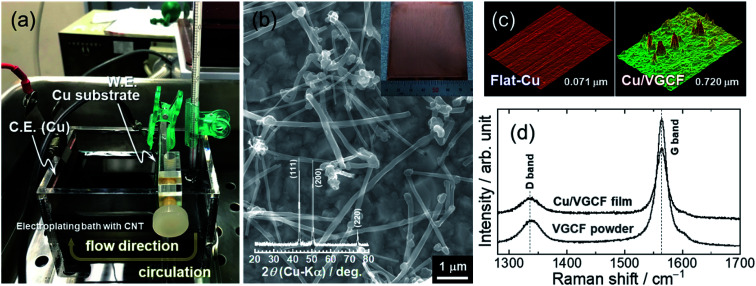
(a) Photograph under preparation for Cu/VGCF film by an electroplating method using an aqueous solution consisted of 0.85 M CuSO_4_ + 0.55 M H_2_SO_4_ with 0.02 mM poly(acrylic acid). (b) FE-SEM images, (c) CLSM image, and (d) Raman spectra of Cu/VGCF composite current collector. Current density and charge amount during electroplating were 5 mA cm^−2^ and 2.7 C cm^−2^, respectively. Inset: XRD pattern of the Cu/VGCF film.

Considering the adhesion strength and conductive pathway between the active material layer and the substrate, it is favorable that CNTs extend inside the electrode mixture based on height direction. We focused on four commercially available MWCNTs: Baytube, MWNT7, VGNF, and VGCF (Fig. 2). The MWCNTs excluding Baytube have few defects and high graphitization degree, and it is expected high electrical conductivity (Fig. S1, ESI†). VGCF (vapor growth carbon fiber) with a diameter of 150 nm or more is rigid and longwise (10–20 μm) compared with other MWCNTs. We therefore used VGCF in preparation of the composite substrate. CNTs aggregate by van der Waals' force and settle out in an aqueous solution due to their own weight (true density: 2.0 g cm^−3^).^33^ In this study, VGCF powder was dispersed by adding PAA and the solution bath was continuously stirred during electroplating (Fig. S2, ESI†). In addition, because the greater the collision frequency between MWCNTs and substrates, the larger the co-deposition amount tends to be, the concentration was set relatively high to 5 g L^−1^. At higher concentrations, there was no significant change in the amount of MWCNT on the Cu surface visually. Fig. 3 shows FE-SEM images, photographs and XRD patterns of the Cu/VGCF composite, displaying that homogenous incorporation of VGCF at the surface was successfully performed by Cu electrodeposition. The composite substrate did not include impurities such as hydroxide and oxides and was composed only of pure metallic Cu. There was no unevenness even over a wide area and it can be observed that each one is deposited independently (Fig. S3, ESI†). Although we cannot measure accurate adhesion, it was immobilized without peeling even when rubbed with tweezers. We are now trying to evaluate the adhesion force quantitatively. Owing to the vertically immobilized VGCF, the surface was roughened and the root-mean-square roughness of the substrate surface (RMS), an index parameter of the degree of irregularity was 10 times larger than a typically used flat-Cu substrate (Fig. 3c). Raman spectra of the substrate surface at any point indicated a signature showing the presence of CNTs: graphite structure-derived G band and defect-induced D band were recognized at 1565 cm^−1^ and 1335 cm^−1^, respectively.^34,35^ The intensity ratio of D band to G band (*I*_D_/*I*_G_), crystallinity affecting electrical conductivity was changed little before (0.219) and after electroplating (0.307) using a strong acid aqueous solution (pH 0.3).

**Fig. 4:**
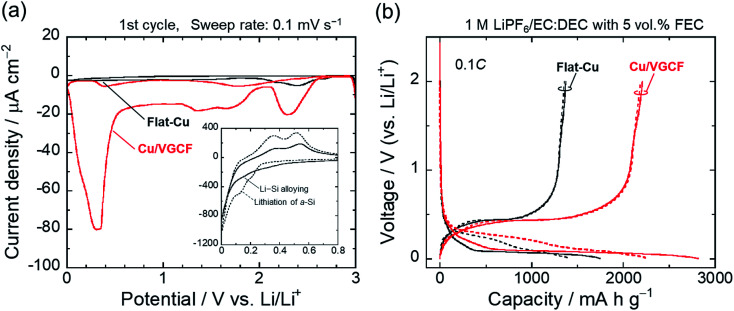
(a) Cyclic voltammograms of Cu/VGCF and flat-Cu substrates in 1 M LiPF_6_/EC : DEC (50 : 50 vol%) with 5 vol% FEC in the potential range of 0.0–3.0 V *vs.* Li/Li^+^. (b) Initial charge–discharge properties of Si electrodes prepared using commercially available Cu and Cu/VGCF substrates (solid line: 1st, dotted line: 2nd cycle).

The electrochemical behavior of the Cu/VGCF composite substrate was evaluated using cyclic voltammetry with a sweep rate of 0.1 mV s^−1^ in the potential range of 0–3.0 V *vs.* Li/Li^+^ (Fig. 4a). In respective systems including the flat-Cu substrate, a broad cathodic peak observed at a relatively high potential of 2.58–2.15 V is attributed to the reductive decomposition of LiPF_6_ involved in the formation of insulating layers mainly composed of LiF.^36^ Reductive current densities at around 1.3 V are due to the decomposition of the electrolyte solvent, and peaks between 1.9−1.5 V and below 0.52 V observed only in the Cu/VGCF substrate are assigned to the irreversible Li intercalation into the graphitic layer and/or channel of CNT,^37–40^ which are also the evidence that VGCF is immobilized on the substrate surface and an electron conduction pathway is fabricated (Fig. S4, ESI†). Diffusion barrier of Li ion inside channel is probably large because the length of VGCF used in this study is relatively lengthwise. Side reactions such as Li trap dependent on surface functional groups and defect sites of CNT.^39,40^ Although the degree of electrolyte decomposition is larger than that of a conventional flat-Cu, it is presumably because of the high surface area originated from VGCF and it is negligible compared with a system with an active material layer. When the active material layer was intentionally peeled from the current collector, it was observed that the extension of VGCF into the electrode mixture was maintained (Fig. S5, ESI†). Fig. 4b exhibits the initial charge–discharge (lithiation/delithiation) profiles of the Si electrodes prepared using the flat-Cu and Cu/VGCF substrates in the voltage range of 0.005–2.0 V (*vs.* Li/Li^+^). In the flat-Cu system, two main voltage plateaus associated with stepwise lithiation reactions were confirmed at 0.27–0.087 V and 0.087–0.005 V,^8,13^ whereas the plateaus were identified at distinctly higher voltages in the Cu/VGCF system (Fig. S6, ESI†). The similar situation is seen in Si electrodes using the embroidered Cu microwire current collector, reported by Brezesinski *et al.*^41^ Spaces formed by three-dimensional Cu network with a 150 nm-diameter can accommodate volume change in Si during lithiation/delithiation. The network mitigated loss of electrical contact of the active material to reduce overvoltage for Li–Si alloying reactions. Initial charge/discharge capacities were 1745/1373 mA h g^−1^ for the flat-Cu and 2816/2207 mA h g^−1^ for the Cu/VGCF. The obvious lower overvoltage makes it clear that the VGCF immobilized on the current collector ensures a good electrical network between the substrate and the electrode mixture to improve the utilization ratio of the active material. The advantage remained after the first cycle. Note that first coulombic efficiencies were comparable (78%), even though the Cu/VGCF-Si electrode showed a larger reversible capacity by 840 mA h g^−1^, which indicated that the VGCF played a key role in anchoring the active material layer to the current collector during the significant volume change in Li–Si alloying/dealloying reactions.

**Fig. 5:**
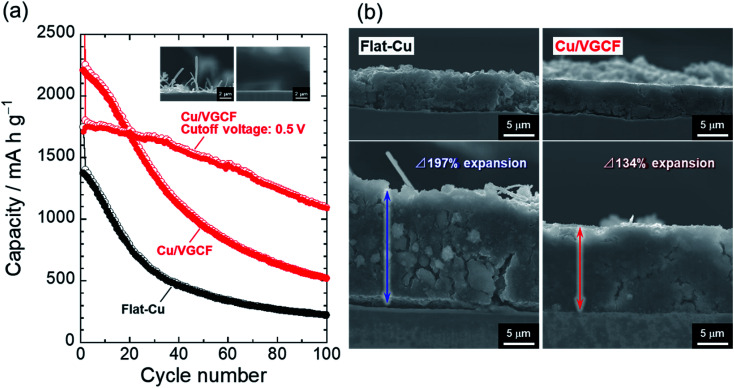
(a) Dependence of charge/discharge capacities on cycle number for Si electrodes operated in the voltage ranges of 0.005–2.00 V and 0.005–0.50 V. (b) Cross-sectional FE-SEM images of the electrodes before and after the 20th cycling in the voltage range of 0.005–0.50 V. Fibers observed at top part of the images are glass separator.

Fig. 5 represents the dependence of charge/discharge capacities on cycle number for Si electrodes. The Cu/VGCF-Si electrode exhibited a larger capacity than that of the flat-Cu–Si electrode but still resulted in poor performance: only a reversible capacity of 520 mA h g^−1^ was maintained after the 100th cycle. Even with the roughened substrate, it was not able to withstand the huge expansion and contraction of Si (Δ280%).^8^ In an effort to suppress the excess volume change, we changed the upper cutoff voltage from 2.0 V to 0.5 V to try to achieve a more stable cycling performance. In that condition, a surface layer formed on the active material thought to be protected from oxidative decomposition at high potential and physical damage.^42^ There was no noticeable effect in the flat-Cu–Si electrode, though the capacity decline was mitigated (Fig. S7, ESI†), whereas the anchor effect and electrical network developed inside the electrode was remarkably revealed: the Cu/VGCF current collector delivered a reversible capacity of approximately 1100 mA h g^−1^ after the 100th cycle. This is not just due to the trade-off between capacity and cyclability under the suppression of volume change. In the Cu/VGCF system, the accumulated capacity after 100 cycles was 1.3 times higher than the condition without the potential control (Fig. S8, ESI†). The thickness of the flat-Cu–Si electrode irreversibly expanded to 197%, and cracks and detachment of the active material layer responsible for the capacity fading were confirmed (Fig. 5b). In contrast, such significant disintegration of the electrode was not observed in the Cu/VGCF system, and the expansion remained a smaller 134%, demonstrating the suppression of electrical isolation due to the anchor effect originating from the VGCF on the substrate surface.

**Fig. 6:**
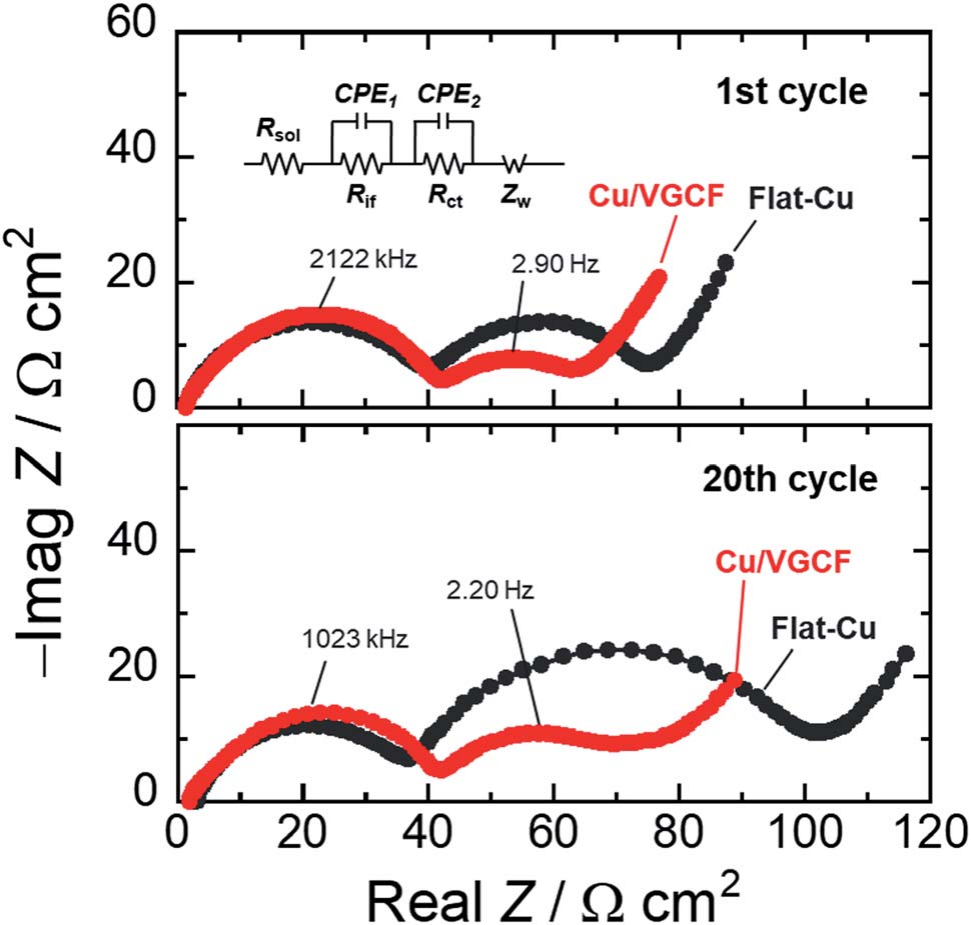
Nyquist plots of asymmetric [Si electrode|Li metal] cell (0.005 V) at the first cycle and after 20th cycling in 1 M LiPF_6_/EC : DEC (50 : 50 vol%) with 5 vol% FEC at potential range of 0.005–0.50 V. Inset: the equivalent circuit for impedance analysis.

Fig. 6 depicts the Nyquist plots of asymmetric [Si electrodes/Li] cells at the first cycle and the 20th cycle operated in the voltage range of 0.005–0.5 V (*vs.* Li/Li^+^), which verifies the significance of developed electron conduction pathways inside Cu/VGCF-Si electrode. The impedance measurement was conducted at the potential of the lower cutoff voltage of 0.005 V, and the apparent areas of the Si electrode and Li foil are 0.79 and 2.0 cm^2^, respectively. We can identify two semicircles and a straight line with a slope of 45° in the high and low frequency regions. The first semicircle comes from the resistance of interfacial Li-ion conduction in a surface layer such as the solid electrolyte interphase (*R*_if_), and the second semicircle denotes the charge transfer resistance associated with lithiation/delithiation reactions (*R*_ct_). The straight line in the low frequency is interpreted as the solid-state diffusion of Li (Warburg impedance: *Z*_w_).^43,44^ Since each size of semicircle observed in the high frequency region is the same degree, it is reasonable that the semicircles can be assigned to the interfacial Li-ion conduction (*R*_if_). In the first cycle, the charge transfer resistance in the Cu/VGCF system was 25 Ω cm^2^ which decreased to 60% of that obtained in the flat-Cu system (41 Ω cm^2^) (Fig. S9, ESI†). The reciprocal of interfacial Li-ion conductivity obeys the Arrhenius equation:^43,45^

1 1/*R*_ct_ = *A* exp(−*E*_a_/*RT*)

where the symbol *A*, *E*_a_, *R*, and *T* are defined as frequency factor, activation energy, gas constant, and absolute temperature, respectively. The decrease in the charge transfer resistance by changing the current collector from the flat-Cu to Cu/VGCF is due to the increase in frequency factor depending on the active site. That is, Si powder inside the electrode directly connects to the VGCF immobilized on the current collector, and the lower resistance also supports the developed electrical network. The fact that the resistance is still smaller after the 20th cycle probably originates from the improved mechanical robustness due to the anchor effect (the suppression of peeling of active material layer). The favorable conduction pathway through the VGCF kept for at least 100 cycles (19 Ω cm^2^).

Further improvement of cycling performance is expected due to not only the physical anchor effect but also introduction of chemical bonding between MWCNT and Si. Optimizing the Si particle size^20,21^ and binder^42,46^ are also effective approaches. In the near future, we are going to report on higher electrode performance based on the formation of strong adhesion due to the chemical modification of MWCNTs.

## Conclusions

Cu/MWCNT composite substrates were prepared with an electroplating technique utilizing co-deposition, and the applicability of the substrate as a current collector of Si electrodes for Li-ion batteries was studied. The crystallinity of the VGCF affecting electrical conductivity in MWCNT was little changed even after electroplating using a strong acid aqueous solution. Si electrodes using the flat-Cu showed only a reversible capacity of 220 mA h g^−1^ after the 100th cycle. On the other hand, the Cu/VGCF substrate delivered a capacity of 520 mA h g^−1^. By controlling the upper cutoff voltage from 2.0 V to 0.5 V, the excess volume change in Si was suppressed to improve cycle stability: a capacity of 1100 mA h g^−1^ with 64% capacity retention was achieved even after the 100th cycle. In the charge/discharge condition, electrode thickness in the flat-Cu system expanded to approximately 200%, whereas the thickness in Cu/VGCF system remained a relatively smaller at 134%, demonstrating the anchor effect originating from the VGCF extending inside the active material layer. EIS measurements revealed that the VGCF functioned as an electron conduction pathway to promote reversibility of lithiation/delithiation reactions. We succeeded in educing the potential high theoretical capacity of Si due to the synergistic effects. Electrode performance should be increased by the introduction of chemical bonding between MWCNT and Si.

## Conflicts of interest

There are no conflicts to declare.

## Supplementary Material

RA-009-C9RA03000J-s001
