# Supplementary material for: Electrodeposited Cu/MWCNT composite-film: a potential current collector of silicon-based negative-electrodes for Li-Ion batteries
Source: RSC Adv. 2019 Jul 15;9(38):21939–45. doi: 10.1039/c9ra03000j (PMC9066439; doi:10.1039/c9ra03000j)
Supplement: RA-009-C9RA03000J-s001 [file RA-009-C9RA03000J-s001.pdf]

Electronic Supplementary Material (ESI) for RSC Advances.
This journal is © The Royal Society of Chemistry 2019

Electronic Supplementary Information (ESI)

Electrodeposited Cu/MWCNT composite-film: a potential current collector of silicon-based negative-electrodes for Li-ion batteries

[a] Department of Materials Chemistry, Faculty of Engineering, Shinshu University

[b] Institute of Carbon Science and Technology, Faculty of Engineering, Shinshu University

4-17-1 Wakasato, Nagano 380-8553, Japan

Masahiro Shimizu,[*ab] Tomonari Ohnuki,[a]
Takayuki Ogasawara,[a] Taketoshi Banno,[a] Susumu Arai[*ab]

Dr. Masahiro Shimizu
E-mail: shimizu@shinshu-u.ac.jp
Tel: +81-26-269-5627; Fax: +81-26-269-5627

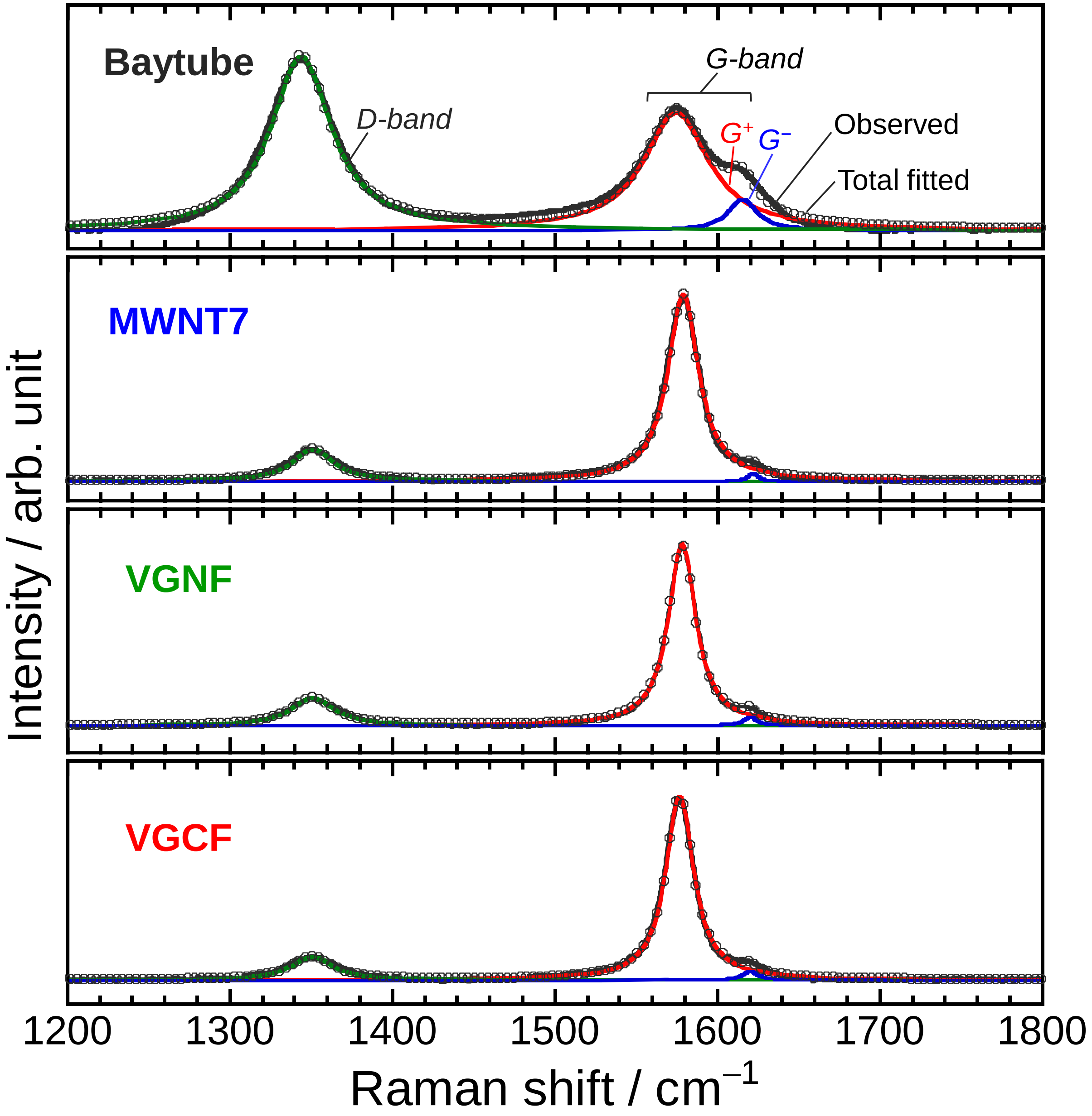

Figure S1. Raman spectra of several MWCNTs. The spectroscopic measurements were conducted through the use of quartz cell at room temperature. G^{+} feature is assigned to in-plane vibration along tube axis. G^{-} feature is originated from in-plane vibration along circumferential direction. Ref.) M. S. Dresselhaus *et al.* *Phys. Rep.* **409** (2005) 47.

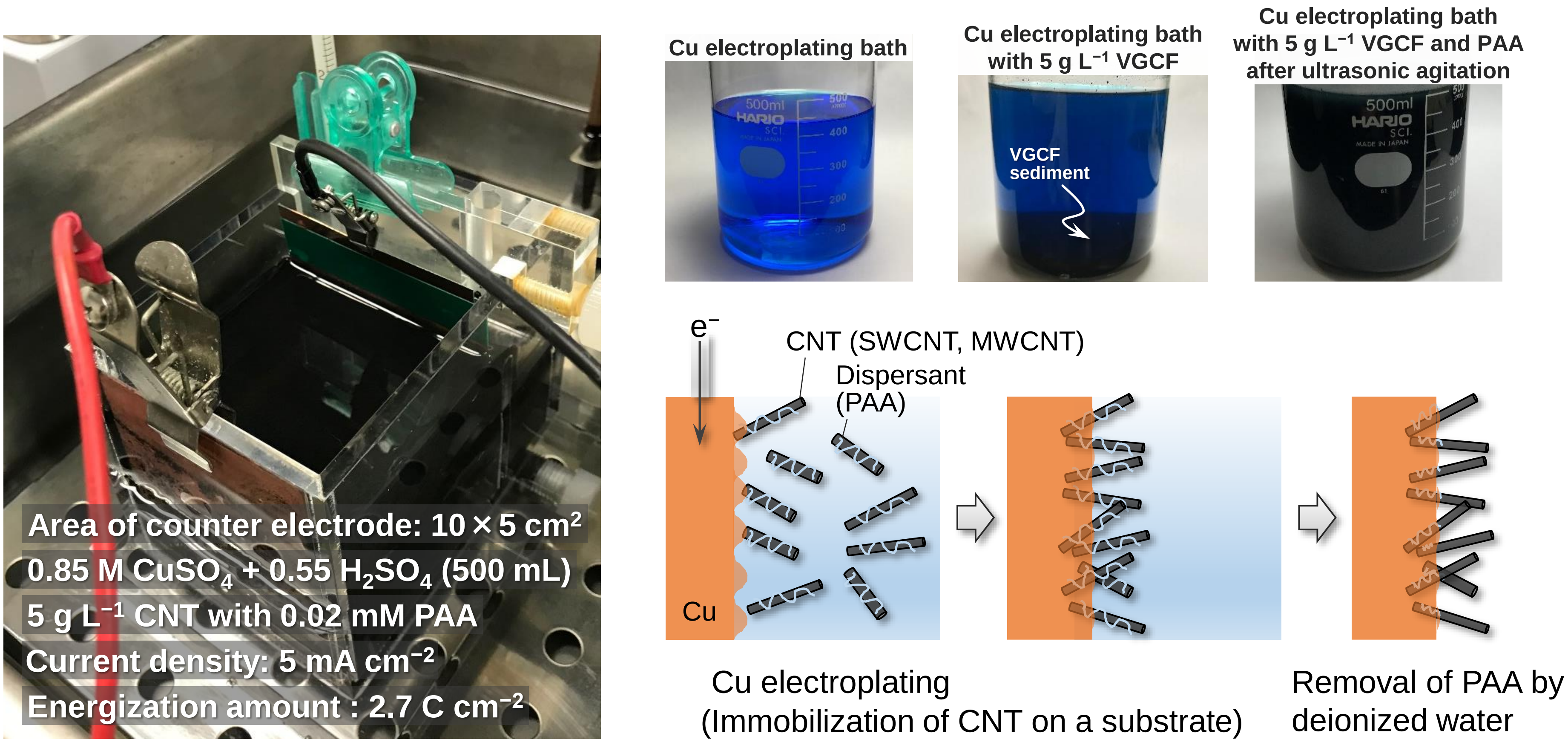

Figure S2. Schematic diagram of formation mechanism for CNT/Cu composite substrate using an electroplating method. Firstly, MWCNT powder was added to an electroplating bath and dispersed homogeneously using 0.02 mM PAA (*Mw*. 5000) followed by ultrasonic homogenizer treatment. Then, a Cu substrate (W.E.) was placed at 6.5 cm from counter electrode (Cu substrate).

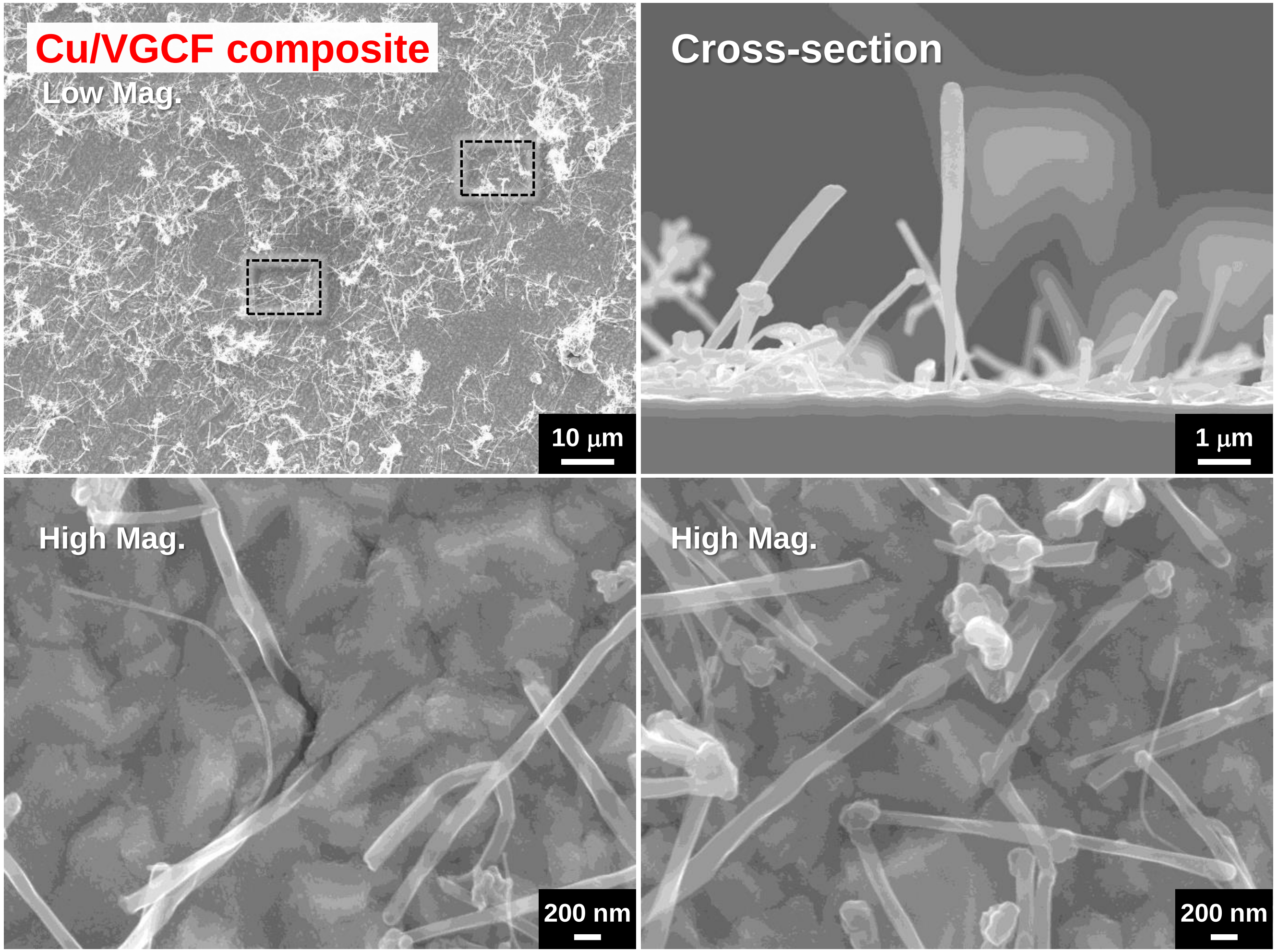

Figure S3. Surface and cross-sectional FE-SEM images of Cu/VGCF composite substrate prepared by an electroplating method. Bottom images demonstrate that MWCNTs are embedded at surface by electroplating-films.

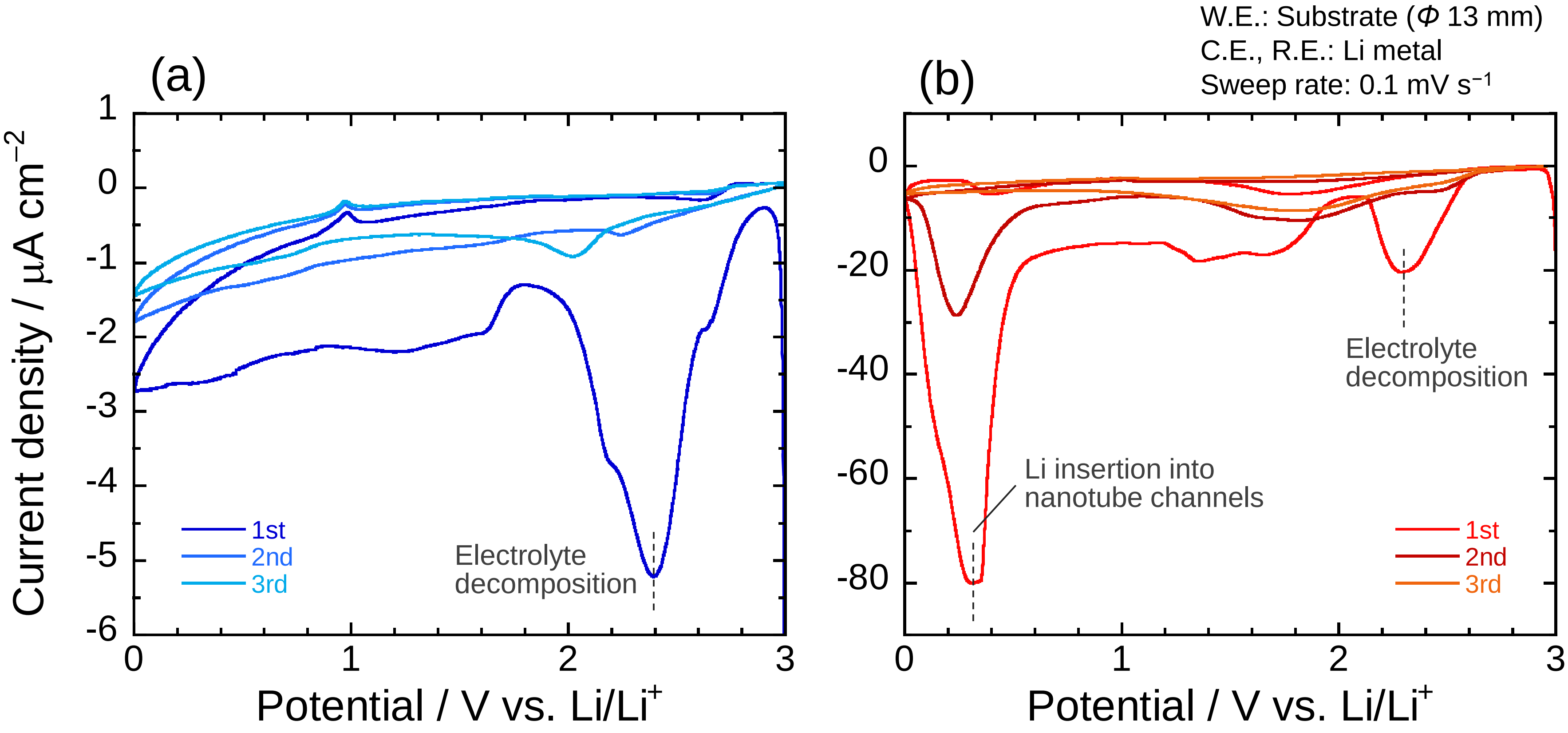

Figure S4. Cyclic voltammograms of current collectors of (a) flat-Cu (commercially available) and (b) Cu/VGCF in 1 M $LiPF_6$/EC:DEC (50:50 vol.%) with 5 vol.% FEC. The reason why the peak intensity for electrolyte decomposition in Cu/VGCF is stronger than that of flat-Cu is probably due to its larger surface area by VGCF (MWCNT). A broad peak observed below 0.15 V vs. Li/Li^+ is attributed to irreversible Li^+ insertion into nanotube channels. Ref) C. M. Schauerman *et al*., *J. Mater. Chem*., **22** (2012) 12008−12015.

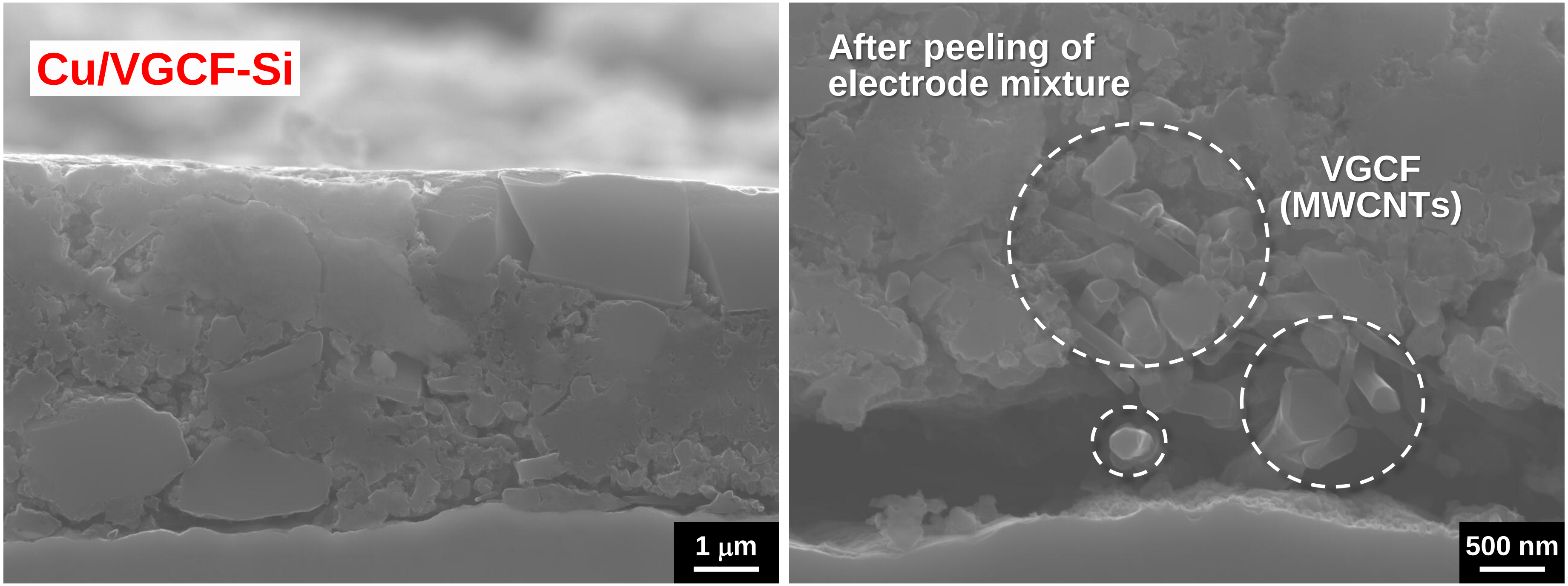

Figure S5. Cross-sectional FE-SEM images of Si electrodes prepared using Cu/VGCF current collector. In order to facilitate visualization of effect for using Cu/VGCF composite, a electrode mixture layer was intentionally peeled off from the current collector by distortive treatment. Fibrous substance observed in the area enclosed by the dotted line is VGCF (MWCNTs). It is clear that VGCF immobilized on the substrate extend inside the active material layer, like a support pillar. The ideal structure contributes the suppression of peeling of the electrode material, even during significant volume change in Si during Li–Si alloying/dealloying reactions.

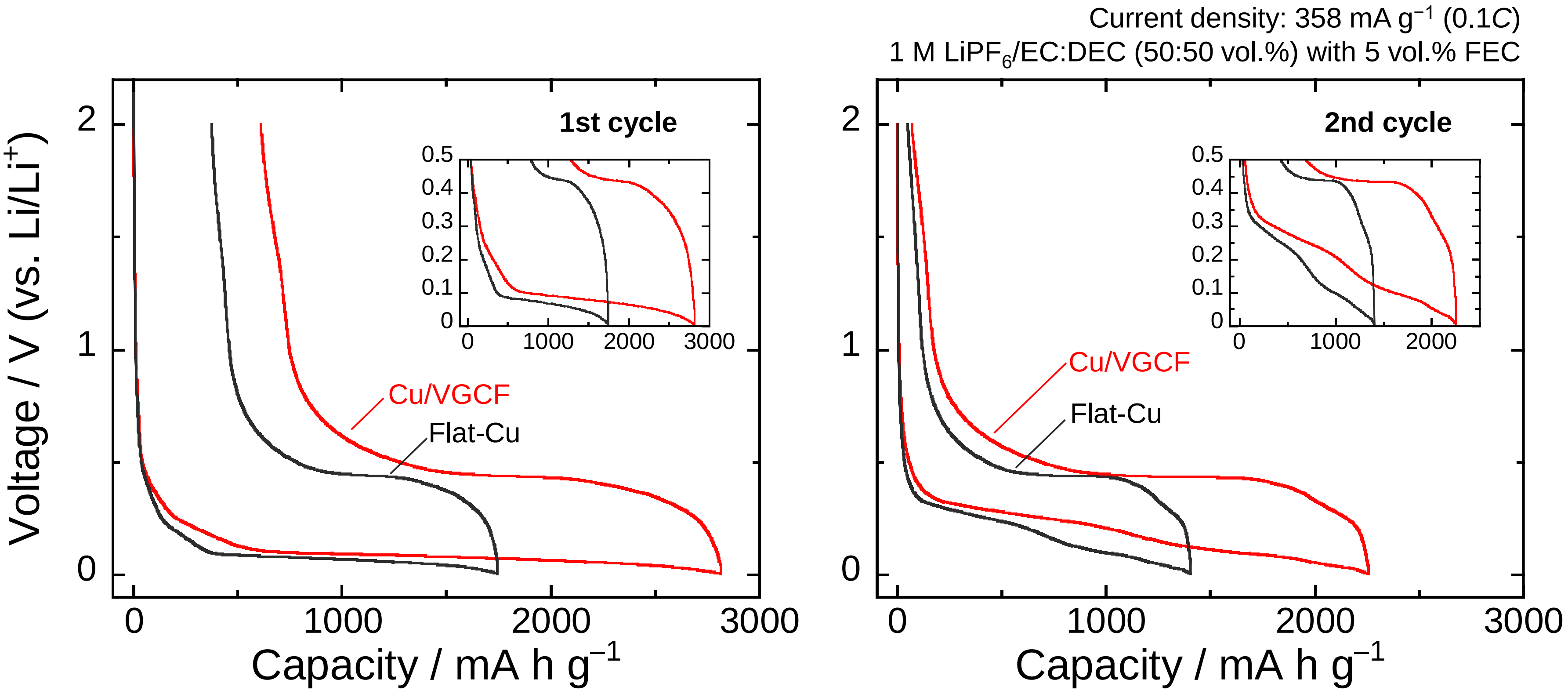

Figure S6. Initial charge−discharge hysteresis curves of Si electrodes prepared using flat-Cu or Cu/VGCF composite substrate (right: 1st, left: 2nd cycle). Overvoltage for lithiation reactions observed below 0.1 V (vs. Li/Li$^+$) in the system with Cu/VGCF substrate is clearly lower than that of flat-Cu system. The favorable phenomenon is also shown at the second cycle. Note that Coulomb efficiencies at the first and second cycles are comparable despite the initial charge capacity in the Cu/VGCF system is larger.

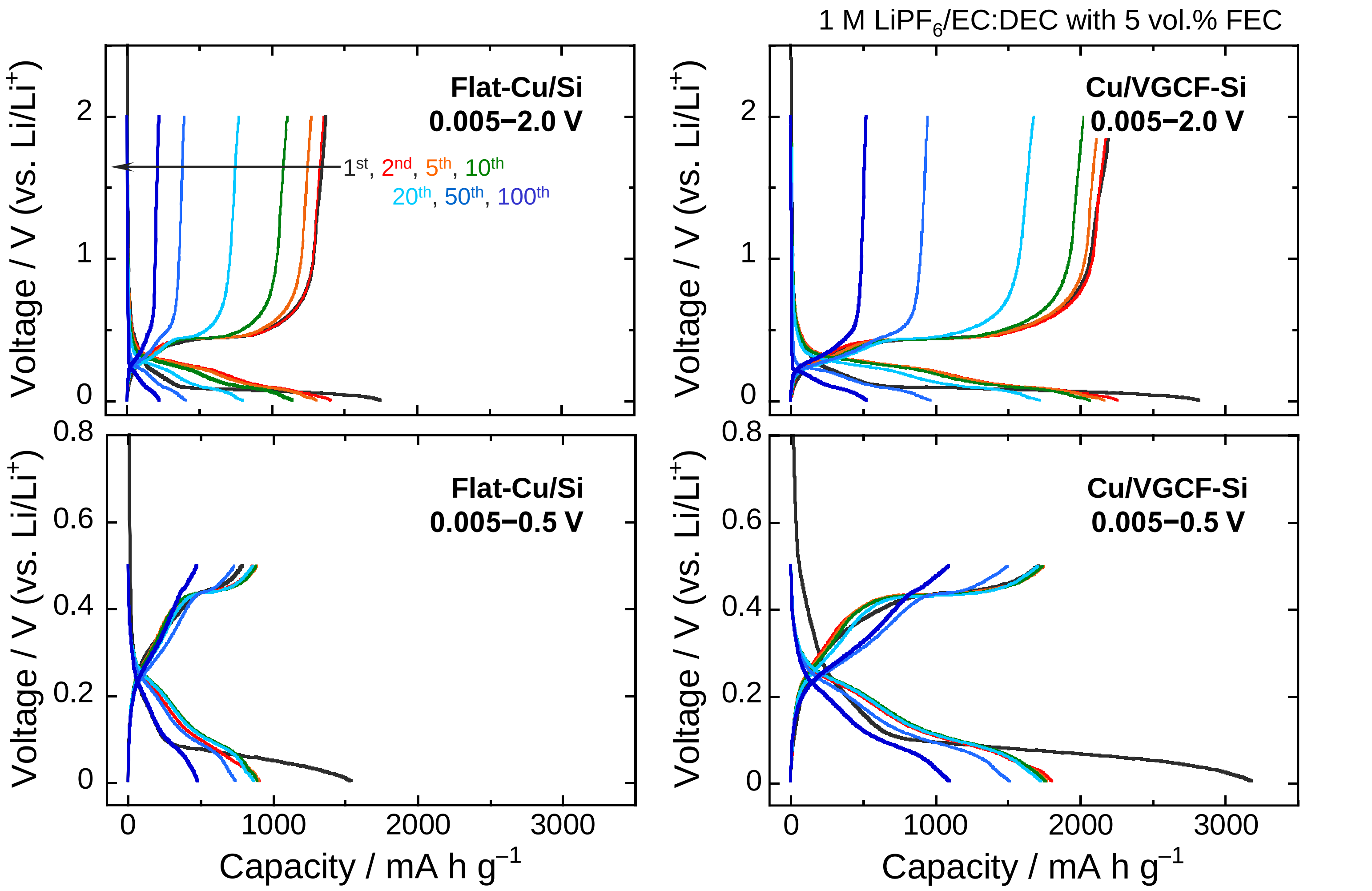

Figure S7. Charge–discharge (Li-insertion/extraction) profiles of Si electrodes with flat-Cu or Cu/VGCF composite in 1.0 M $LiPF_6$/EC:DEC (50:50 vol.%) under a constant current density of 358 mA g^{-1} (0.1*C*). The upper cut-off voltage was changed from 2.0 V to 0.5 V (vs. Li/Li^{+}) to suppress excess volume expansion of Si during Li–Si alloying reactions.

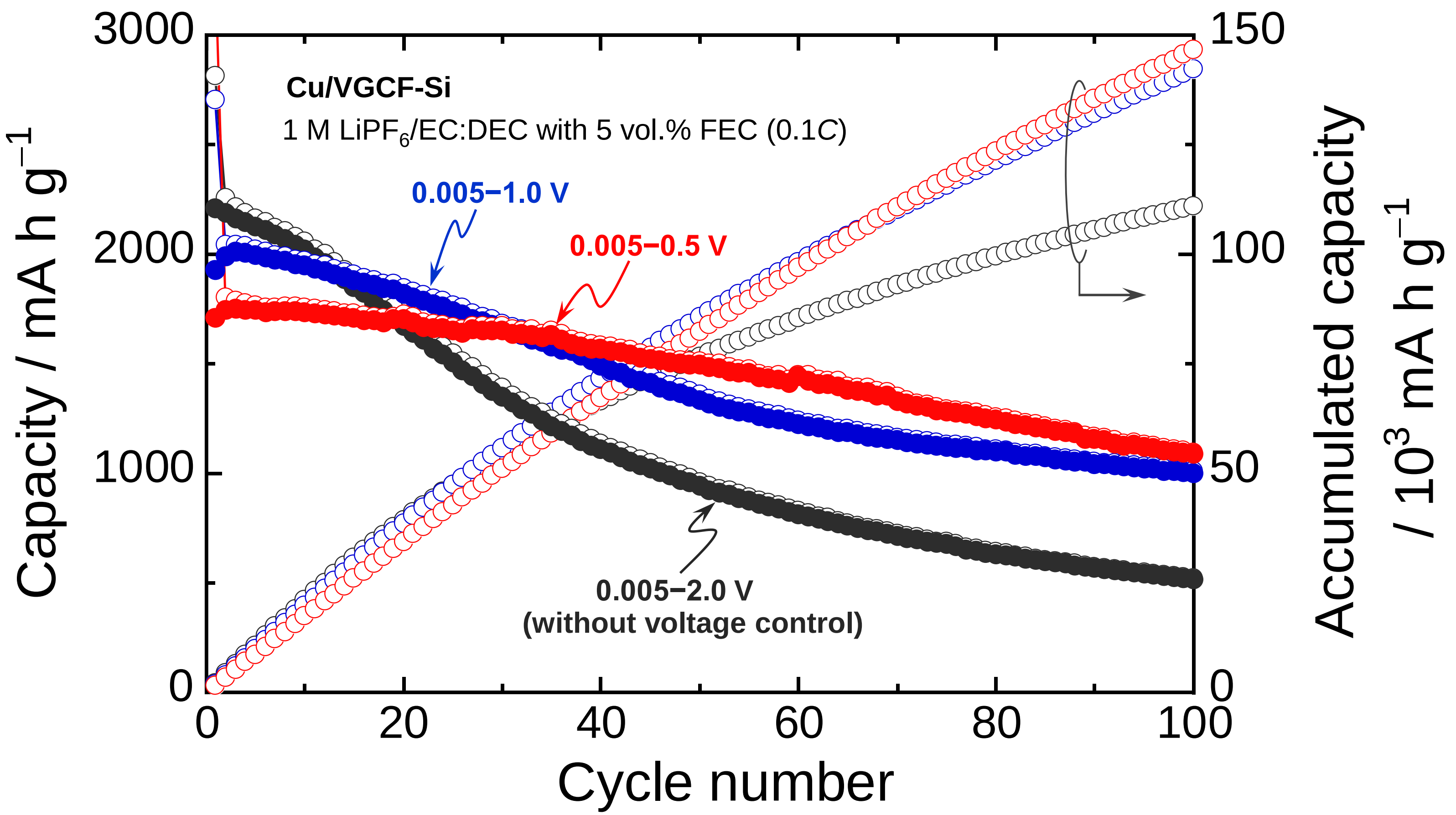

Figure S8. Influence of upper cutoff voltage on cycle stability of Si electrode prepared using Cu/VGCF current collector. Electrolyte: 1 M $LiPF_6$/EC:DEC with 5 vol.% FEC, Current density: 358 mA g^{-1} (0.1*C*). The best cyclability was achieved by the adjusting 0.5 V as the upper cutoff voltage. Accumulated capacity obtained in the voltage range of 0.005–0.5 V was largest.

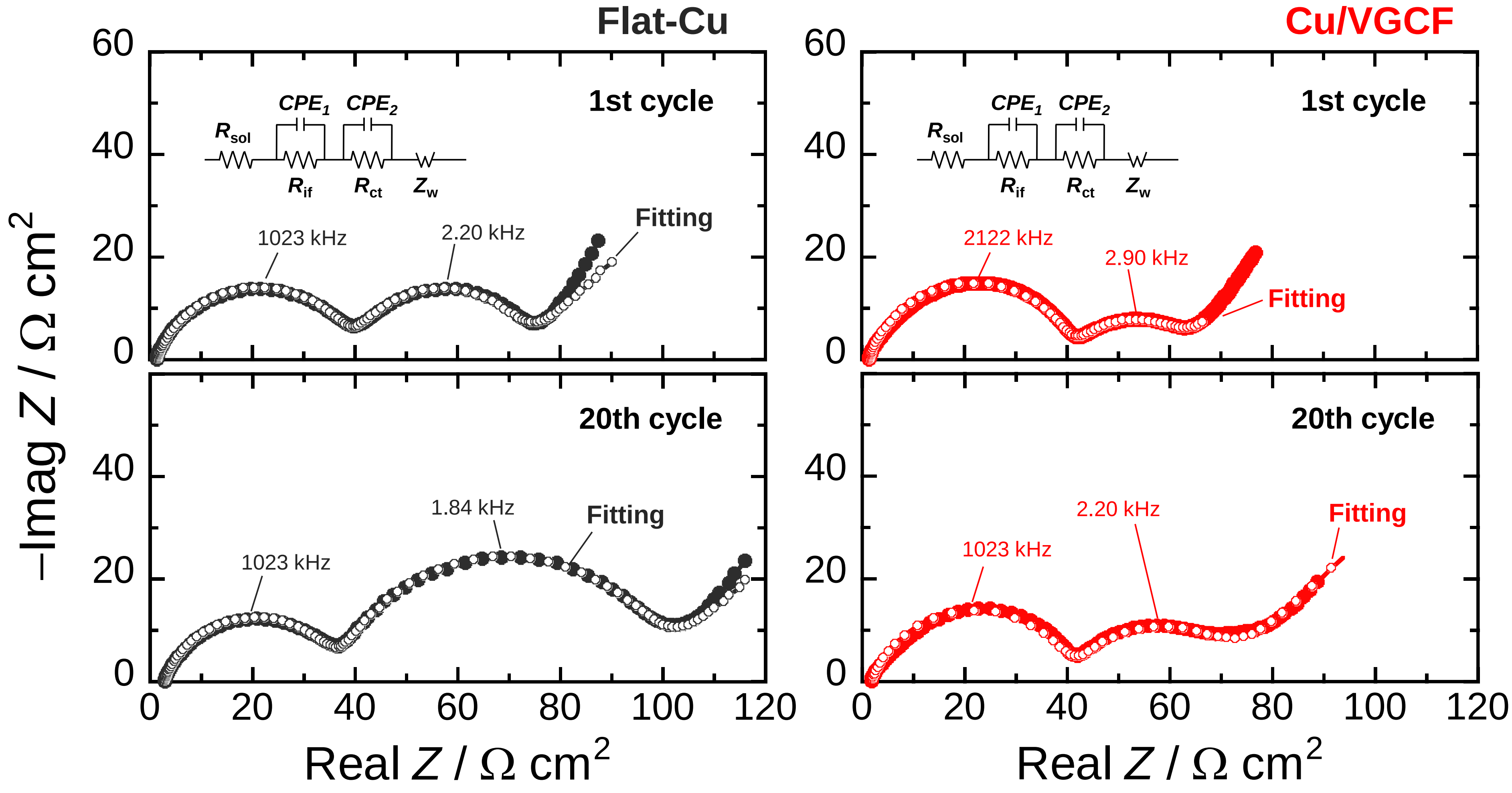

Figure S9. Nyquist plots of asymmetric [Si electrode | Li metal] cells at 298 K. The closed circuits is fitting results calculated by using Randles circuit. Open and closed circles are measured value and fitting results, respectively.
